# Contemporary Dissemination Rates Among Research Presented at Obstetrics and Gynecology Subspecialty Conferences

**DOI:** 10.1097/og9.0000000000000113

**Published:** 2025-09-18

**Authors:** Minhazur R. Sarker, Maha Pasha, Blair Thompson, Megan King, Dylan Hutson, Rachel Wiley, Nika Kunwar, Dana Canfield, Sierra Adkins, Harriet Rothschild, Gladys A. Ramos, Cynthia Gyamfi-Bannerman

**Affiliations:** Department of Obstetrics, Gynecology, and Reproductive Sciences, University of California, San Diego, and the University of California San Diego School of Medicine, La Jolla, California.

## Abstract

**OBJECTIVE:**

We aimed to evaluate the conversion rate of conference abstract presentation to full manuscript among obstetrics and gynecology subspecialty society meetings.

**METHODS:**

We conducted a retrospective cohort study of 2019 meeting supplements from four subspecialty societies: 1) the American Society for Reproductive Medicine (ASRM), 2) the American Urogynecologic Society (AUGS), 3) the Society of Gynecologic Oncology (SGO), and 4) the Society for Maternal-Fetal Medicine (SMFM). All abstracts that were not subsequently withdrawn were included and queried on PubMed using a multimodal method that incorporated abstract keywords and the names of the first and last author to determine publication status. An abstract was classified as a published manuscript if the first author of the meeting abstract was credited in the final publication. The primary outcome was overall publication status. Secondary outcomes included subdivided oral and poster abstract publication conversion, publication journal type, manuscript journal impact factor, time to publication, and additional presentation metrics.

**RESULTS:**

Among the 3,425 total conference abstracts presented in 2019, the overall oral and poster abstract publication rate for all societies combined was 39.7% (subspecialty range, 33.1–47.5%). For only oral abstracts, 61.0% (subspecialty range, 50.7–78.6%) were published and, for only poster abstracts, 35.4% (subspecialty range 27.1–45.4%) were published. When comparing among subspecialty societies, there were significant differences in rates of oral abstract conversion, with ASRM at 50.7%, AUGS at 64.9%, SGO at 78.6%, and SMFM at 74.5% (*P*<.01), and in rates of poster abstract conversion, with ASRM at 27.1%, AUGS at 27.3%, SGO at 45.4%, and SMFM at 40.5% (*P*<.01). Most of the dissemination occurred in general obstetrics and gynecology journals and not in the society associated journals. The median (interquartile range) journal impact factor was 4.7 (2.1, 7.2) among oral abstracts and 3.3 (2.1, 4.8) among poster abstracts (*P*<.01). The median (interquartile range) time to publication was 16 months (9, 26 months) among oral abstracts and 17 months (9, 30 months) among poster abstracts (*P*=.13).

**CONCLUSION:**

Our contemporary evaluation of women's health research dissemination shows that a significant portion of research presented at obstetrics and gynecology subspecialty conferences remains unpublished, even after 5 years.

## Abstract 2

Five years after presentation at obstetrics and gynecology subspecialty conferences, more than one-third of oral abstracts and more than one-half of poster abstracts remain unpublished in peer-reviewed journals.

Conducting research is crucial to the advancement of medical knowledge; the dissemination of research findings, both positive and negative, allows the larger medical community the opportunity to change practice, behavior, and future development. Although the publication of peer-reviewed research has skyrocketed in the past decade, research presented at conferences in the form of research abstracts often does not transition easily to the scientific community at large.^1^ There are limitations to conference abstracts, including a truncated review compared with full-length manuscripts, lack of details on methods and adherence to guidelines such as CONSORT (Consolidated Standards of Reporting Trials), and scant nuance in data analysis and interpretation.^2,3^ Additionally, conference abstracts are less available to the scientific community, and unusual or negative trials often remain unpublished.^4^ With the goal of disseminating quality research, the gold standard remains peer-reviewed publication.

Across numerous medical specialties, there is great variance in the conversion of conference abstracts to manuscripts, ranging from 29.3% to 69.1%, depending on whether the conference abstract was an oral or poster presentation.^5–26^ Within the field of obstetrics and gynecology, conference abstracts exhibit a similar trend; however, the majority of these published studies were from the 1990s and early 2000s, with few studies conducted in a more contemporary timeframe.^6,17,20,22^ Even fewer studies have examined the rate of manuscript conversion from poster presentations.

No studies have combined multiple subspecialties within obstetrics and gynecology to provide a more global state of women's health research dissemination. We determined the conversion rate from contemporary conference abstracts to manuscripts among oral and poster presentations across the major subspecialty conferences within obstetrics and gynecology.

## METHODS

We collected data from the 2019 annual conference meeting supplements from four subspecialty societies: 1) the American Society for Reproductive Medicine (ASRM), 2) the American Urogynecologic Society (AUGS), 3) the Society of Gynecologic Oncology (SGO), and 4) the Society for Maternal-Fetal Medicine (SMFM). We evaluated conferences from 2019 to allow a 5-year time interval from the meeting to publication. We included any abstract published in the respective subspecialty society journals: *Fertility and Sterility* (ASRM), the *International Urogynecologic Journal* (AUGS), *Gynecologic Oncology* (SGO), and the *American Journal of Obstetrics & Gynecology* (SMFM).^27–30^ Each conference meeting had different categories of abstracts, which were subsequently classified into oral or poster categories. ASRM and SMFM had standard oral and poster abstracts. AUGS had two oral types (long format and short format) and two poster types (scientific salon and routine poster). SGO had standard oral abstracts and four poster types (late-breaking, featured, special interest, and routine). We excluded all abstracts that were withdrawn or retracted, because the data were not available to be searched.

Each published conference abstract was manually searched on PubMed between April and December 2024 to determine publication conversion to research manuscript using a multimodal method that encompassed a combination of the first and last authors' names, the conference abstract title, and keywords from the conference abstract (eg, names of medications, disease of interest, or primary outcome). To be considered published, the first author of the conference abstract must have been listed as an author on the final manuscript. Initial manuscript conversion was assessed by a single reviewer. If multiple conference abstracts were published into one manuscript, all the corresponding conference abstracts were considered published. If there was uncertainty regarding a publication or whether multiple abstracts were compiled into one manuscript, a second reviewer (M.R.S.) independently performed the same search.

The primary outcome was the rate of overall conference abstract conversion (both oral and poster) to manuscript publication among all included subspecialty society conferences. Secondary outcomes included individual oral and poster abstract conversion rates, breakdown of published manuscripts by subspecialty conference, conversion rates for each subspecialty society, the location of publication, first author consistency, journal median current impact factors from 2023, and abstract time to publication in months. The time to publication was further categorized into time intervals from the date of the meeting: before presentation, within 12 months, 13–24 months, 25–36 months, 37–48 months, or more than 48 months. Publication journal impact factor was also categorized as high (4 or higher) or low (lower than 4). Additional descriptive characteristics were collected for the total number of abstracts presented at each conference, the proportion of conference abstracts awarded oral presentation, the breakdown of study designs, the number of authors per abstract, the current highest degrees of the first and last authors, and whether the first and last authors resided in the United States.

Descriptive characteristics and all outcomes were compared using χ^2^, analysis of variance, or Kruskal-Wallis tests, as statistically appropriate. We predefined statistical significance as a *P*-value <.05. Categorical variables are reported as the number of events and precent, and continuous variables are reported as either mean and SD or median and interquartile range. All statistical analyses were performed using STATA IC 15.1. Because public data were used for our study, with no human subject involvement, this study did not require IRB oversight.

## RESULTS

**Fig. 1.**
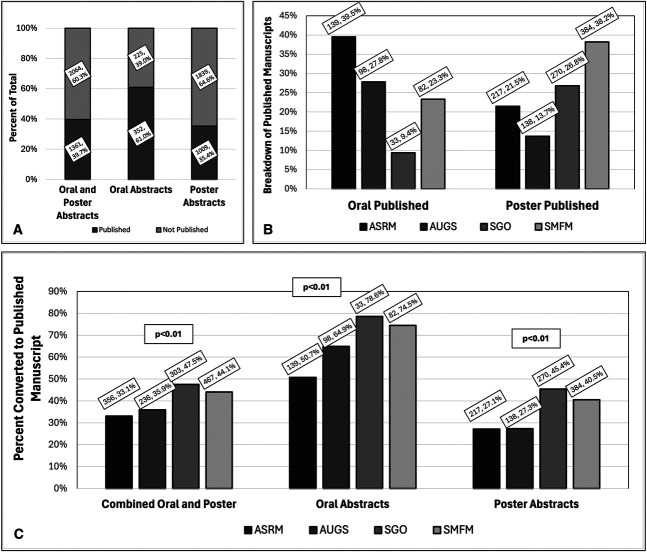
Publication conversion among 2019 subspecialty society meetings. **A**. Percentage of oral and poster abstracts converted to manuscript publication among all societies combined. **B**. Breakdown of published manuscripts by subspecialty society, highlighting relative contribution of each society to the total published group in **A**. **C**. Abstract-to-manuscript conversion rate of each society among combined oral and poster, oral, and poster abstracts. All data labels formatted as n, %. ASRM, American Society for Reproductive Medicine; AUGS, American Urogynecologic Society; SGO, Society of Gynecologic Oncology; SMFM, Society for Maternal-Fetal Medicine.

In 2019, there were a total of 3,425 abstracts presented, with the following breakdown: 1,074 (31.4%) at ASRM, 657 (19.2%) at AUGS, 637 (18.6%) at SGO, and 1,057 (30.9%) at SMFM. There were significant differences in the proportion of abstracts selected for oral presentation at each conference, with 274 (25.5%) at ASRM, 151 (23.0%) at AUGS, 42 (6.6%) at SGO, and 110 (10.4%) at SMFM (data not shown, *P*<.01). The overall publication rate of oral and poster abstracts among all subspecialty societies combined was 39.7% (subspecialty range 33.1–47.5%), with 61.0% (subspecialty range 50.7–78.6%) for oral abstracts and 35.4% (subspecialty range 27.1–45.4%) for poster abstracts (Fig. 1A and C).

*Sarker. Dissemination of Obstetrics and Gynecology Research. O&G Open 2025*.

Among the 352 oral abstracts converted to manuscript publication, 139 (39.5%) were from ASRM, 98 (27.8%) were from AUGS, 33 (9.4%) were from SGO, and 82 (23.3%) were from SMFM (Fig. 1B). Among the 1,009 poster abstracts converted to manuscript publication, 217 (21.5%) were from ASRM, 138 (13.7%) were from SGO, 270 (26.8%) were from AUGS, and 384 (38.2%) were from SMFM (Fig. 1B). Among subspecialties, there were differences in rates of oral abstract conversion, with ASRM at 50.7%, AUGS at 64.9%, SGO at 78.6%, and SMFM at 74.5%. There also were differences in rates of poster abstract conversion, with ASRM at 27.1%, AUGS at 27.3%, SGO at 45.4%, and SMFM at 40.5% (Fig. 1C, *P*<.01). Among oral abstract conversion, the ASRM rate was statistically significantly lower than SGO or SMFM, and, among poster abstract conversion, the ASRM and AUGS rates were statistically significantly lower than SGO or SMFM (all *P*<.001); otherwise differences were not statistically significant (Fig. 1C).

**Fig. 2.**
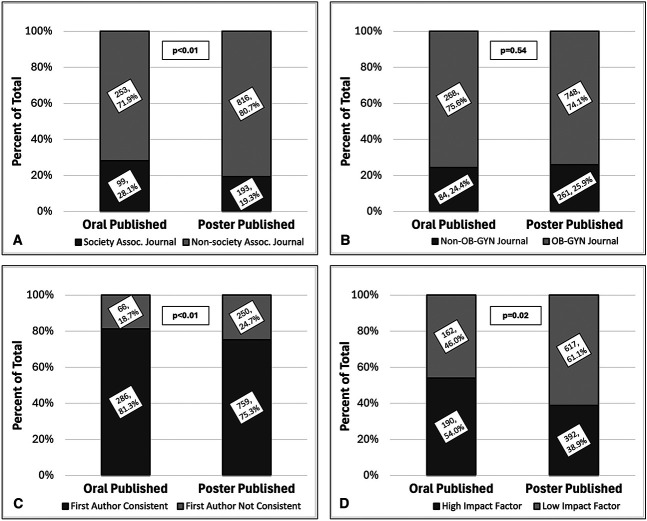
Publication metrics among all abstracts converted to manuscripts from 2019 subspecialty society meetings (n=352 for oral published and n=1,009 for poster published). **A**. Percentage published in each society's respective associated journal (also consistent with fast-track option). **B**. Percentage published in non–obstetrics and gynecology journals vs obstetrics and gynecology journals. **C**. Percentage published with abstract first author remaining consistent with manuscript first author. **D**. Percentage published in high impact factor journal (impact factor of 4 or higher) or low impact factor journal (impact factor lower than 4) journal. All data labels formatted as n, %. OB-GYN, obstetrics and gynecology.

The number of conference abstracts eventually published as manuscripts in subspecialty society-associated journals was 99 (28.1%) for oral abstracts and 193 (19.3%) for poster abstracts (*P*<.01, Fig. 2A). Although most publications occurred in obstetrics and gynecology journals, 84 (24.4%) oral abstracts and 261 (25.9%) poster abstracts were published in non–obstetrics and gynecology journals (*P*=.54, Fig. 2B). Most conference abstracts that were subsequently published maintained consistency in the first author; this was slightly higher for oral conference abstracts (oral 286 [81.3%] vs poster 759 [75.3%]; *P*=.02; Fig. 2C).

*Sarker. Dissemination of Obstetrics and Gynecology Research. O&G Open 2025*.

The median (interquartile range) journal impact factor was 4.7 (2.1, 7.2) for published oral abstracts and 3.3 (2.1, 4.8) for published poster abstracts (*P*<.01, Table 1). Published manuscripts from oral conference abstracts were more likely to be published in high impact factor journals (oral, 190 [54.0%] vs poster, 392 [38.9%]; *P*<.01; Fig. 2D). There was no statistically significant difference between median [interquartile range] time for oral abstracts to be published (16 months [9, 26 months]) compared with poster abstracts (17 months [9, 30 months]) (*P*=.13, Table 1). Time to publication was further categorized into yearly intervals, and oral abstracts appeared to be published slightly earlier than poster abstracts (Table 1, *P*=.03).

**Table 1.** Additional Outcomes Among Published Abstracts From 2019 Subspecialty Society Meetings

Outcome	Oral Abstracts (n=577)	Poster Abstracts (n=2,848)	*P*
Published	352 (61.0)	1,009 (35.4)	<.01
2023 journal impact factor	4.7 (2.1, 7.2)	3.3 (2.1, 4.8)	<.01
Months to publication	16 (9, 26)	17 (9, 30)	.13
Time to publication (mo)			.03
Before presentation	16 (4.6)	62 (6.2)	
Within 12	121 (34.4)	309 (30.8)	
13–24	118 (33.5)	294 (29.3)	
25–36	59 (16.8)	179 (17.9)	
37–48	21 (6.0)	97 (9.7)	
49–60	17 (4.8)	44 (4.4)	
More than 60	0 (0.0)	17 (1.7)	

Data are n (%) or median (interquartile range) unless otherwise specified.

Randomized controlled trials were more often awarded oral presentations (21.5% of all oral abstracts were trials, compared with 4.8% of all poster abstracts, *P*<.01, data not shown), and retrospective cohort studies were more often awarded poster presentations (26.5% of all oral abstracts were retrospective studies, compared with 47.1% of all poster abstracts, *P*<.01, data not shown). Randomized controlled trials were also more likely to be published among oral abstracts (73.4% for trials vs 61.0%, *P*<.01, data not shown) and poster abstracts (46.3% vs 35.4%, *P*<.01, data not shown) abstracts. Other than randomized controlled trials, no other statistically significant differences were noted among study designs and publication conversion. When analyzing the first and last authors’ currently held highest degrees, individuals with an isolated master's degree were less likely to have converted their abstract to manuscript publication. Otherwise, no consistent pattern was present amongst other degrees (Table 2). Finally, there were no statistically significant differences noted with respect to publication conversion and whether first or last authors reside in the United States.

**Table 2.** Characteristics of Oral and Poster Abstracts, Stratified by Publication Status

Characteristic	Oral Abstracts (n=577)			Poster Abstracts (n=2,848)		
	Published (n=352)	Not Published (n=225)	*P*	Published (n=1,009)	Not Published (n=1,839)	*P*
Total abstracts						
ASRM (n=1,074)	139 (39.5)	135 (60.0)		217 (21.5)	583 (31.7)	
AUGS (n=657)	98 (27.8)	53 (23.6)		138 (13.7)	368 (20.0)	
SGO (n=637)	33 (9.4)	9 (4.0)		270 (26.8)	325 (17.7)	
SMFM (n=1,057)	82 (23.3)	28 (12.4)		384 (38.2)	563 (30.6)	
Study design			<.01			<.01
Randomized trial	91 (25.9)	33 (14.7)	<.01	63 (6.2)	73 (4.0)	<.01
Prospective cohort	80 (22.7)	42 (18.7)	.24	234 (23.2)	458 (24.9)	.31
Retrospective cohort	84 (23.9)	69 (30.7)	.07	477 (47.3)	863 (46.9)	.86
Basic science	46 (13.1)	40 (17.8)	.12	114 (11.3)	203 (11.0)	.83
Other	51 (14.5)	41 (18.2)	.23	121 (12.0)	242 (13.2)	.37
No. of authors	6.2±2.4	5.7±2.4	.01	5.7±2.4	5.5±2.3	.03
1^st^ author’s current highest degree			.04			<.01
MD or DO	242 (68.8)	132 (59.7)	.01	726 (72.1)	1,298 (70.7)	.44
MD or DO plus Masters	26 (7.4)	12 (5.4)	.33	77 (7.7)	100 (5.4)	.02
MD, PhD	23 (6.5)	18 (8.1)	.50	60 (6.0)	87 (4.7)	.16
PhD	39 (11.1)	29 (13.1)	.51	105 (10.4)	191 (10.4)	.99
Masters	10 (2.8)	15 (6.8)	.03	20 (2.0)	58 (3.2)	<.01
Bachelors	12 (3.4)	15 (6.8)	.07	0 (0.0)	5 (0.3)	.07
1^st^ author in United States	271 (77.0)	169 (75.1)	.61	644 (63.8)	1,235 (67.2)	.07
Last author’s current highest degree			<.01			.21
MD or DO	238 (68.8)	136 (63.0)	.08	614 (62.3)	1,116 (62.7)	.93
MD or DO plus Masters	18 (5.2)	5 (2.3)	.08	93 (9.4)	131 (7.4)	.05
MD, PhD	32 (9.3)	26 (12.0)	.34	140 (14.2)	229 (12.9)	.28
PhD	54 (15.6)	36 (16.7)	.83	117 (11.9)	253 (14.2)	.10
Masters	2 (0.6)	10 (4.6)	<.01	6 (0.6)	9 (0.5)	.36
Bachelors	2 (0.6)	3 (1.4)	.33	3 (0.3)	9 (0.5)	.71
Last author in United States	265 (76.6)	165 (76.4)	.96	640 (64.8)	1,207 (68.0)	.18

ASRM, American Society for Reproductive Medicine; AUGS, American Urogynecologic Society; SGO, Society of Gynecologic Oncology; SMFM, Society for Maternal-Fetal Medicine.

Data are n (%) or mean±SD unless otherwise specified.

Similar descriptive characteristics for conference oral and poster abstracts stratified by subspecialty society are provided in Appendices 1 and 2 (available online at http://links.lww.com/AOG/E323).

## DISCUSSION

We found that more than half of oral abstracts are published, whereas most poster abstracts remain unpublished 5-years postconference. There were differences noted among both oral and poster abstracts when publication rates were stratified by the presentation society. Whether these are affected by pressure to publish, mentorship or guidance, or differences in research dissemination pathways warrants further study. We also identified an inverse relationship between the proportion of abstracts awarded oral presentations and the rate of publication conversion, which suggests that accepting more abstracts for oral presentation may result in lower quality and, thus, lower likelihood of subsequent publication. As evidenced by the increased publication rate among oral abstracts and the associated higher impact factors of those publications, societies are likely accurately distinguishing the quality of abstracts being submitted for consideration.

Regardless of the type of conference presentation, there is considerable research that does not eventually disseminate into the medical literature. Among oral presentations, 39.0% (subspecialty society range 21.4–49.7%) were never published, and this number is much higher, at 64.6% (subspecialty society range 54.6–72.9%), among poster presentations. Not surprisingly, most of the dissemination occurs among obstetrics and gynecology journals with oral abstracts disseminating into higher impact factor journals. Noticeably, time to eventual dissemination did not differ between oral and poster abstracts, and most dissemination occurred within 24 months.

One of the largest studies published analyzing conference abstract to publication rate evaluated 79 meetings across multiple specialties and found an overall publication rate of 44.5%, with increased likelihood of publication for oral abstracts, randomized controlled trials, and studies with positive findings.^5^ Numerous studies that analyzed conference abstract publication rates across various medical subspecialties have noted publication rates to be 29.3–69.1% overall, 40.3–82.4% for oral abstracts, and 36.5–41.4% for poster abstracts.^5,6,8–12,14–17,19–21,23–26^ Compared with the existing literature, the publication rates noted for obstetrics and gynecology subspecialty conferences in 2019 are similar for oral abstract publication rate, but on the lower range for both overall and poster abstracts publication rate.

Although there have been many studies that assessed conference abstracts publication rates, only a few studies have evaluated obstetrics and gynecology subspecialty societies. Only SGO and SMFM are reported on, with no prior studies evaluating ASRM or AUGS. One study analyzed SGO oral abstracts and found that 57.3% were published in 2017 and 58.6% were published 2018, although this study analyzed a publication window of 3 years, instead of 5 years.^20^ Given the increased 5-year publication window, it is not surprising that our results show that the 2019 SGO oral abstract publication rate was 78.6%.

One study analyzed the publication rate of clinical trials presented at SMFM from 2000 to 2002 regardless of oral or poster presentation and found that 55.6% were published.^22^ Our contemporary analysis of 2019 subspecialty societies combined shows a similar overall randomized clinical trial publication rate of 59.2%. Subsequently, another study in 2015 aimed to evaluate the SMFM abstract publication rate from 2003 to 2010 of both oral and poster abstracts over a 5-year period.^6^ They reported a higher overall publication rate (54.3%), oral publication rate (77.1%), and poster publication rate (48.8%) relative to our findings for both the 2019 SMFM conference publication rates and the cumulative publication rates of all subspecialty societies in 2019. They also reported a median time to publication of 11 months for oral abstracts and 21 months for poster abstracts, which is faster for oral abstracts and slower for poster abstracts relative to our findings.^6^

Our contemporary findings highlight lower publication rates in the setting of more obstetrics and gynecology journals available for dissemination. Currently, there are 210 obstetrics and gynecology journals, of which 29 (21 English and eight non-English) were started between 2009 and 2015, 25 (17 English and eight non-English) between 2015 and 2020, and 44 (32 English and 12 non-English) after 2020. This correlates to the additional journals available between the timing of the prior studies noted above.^6,20,22,31^ Although these newer journals may have originated to keep up with the current pace of research output, the majority are lower and volume and have lower impact factors.

Our findings highlight that a significant portion of oral abstracts and poster abstracts presented at obstetrics and gynecology subspecialty conferences remain unpublished as long as 5 years postpresentation. Over the years, the number of abstracts presented annually has steadily increased, and this may reflect the correlation between presentation and conference attendance.^6^ Additionally, given our contemporary analysis, the effect of the coronavirus disease 2019 (COVID-19) pandemic on research follow through, study completion, and eventual dissemination remain poorly understood. Despite shifts in preferential publication of COVID-19 literature during the pandemic, a subsequent 3-year postpandemic window should be sufficient for eventual dissemination of non–COVID-19 research. Aside from increased publication associated with randomized trials, our findings did not show any significant patterns for publication among first or last authors’ degrees or location in the United States or internationally. Higher publication rates for randomized trials also may reflect access to external funding allowing resources for completion of revision analyses or manuscript writing.

Clinicians who assess conference abstracts should consider maintaining a level of skepticism if the research remains unpublished over the coming years. Although it is not feasible to revise conference supplements after publication, it should be standard to maintain caution with respect to conclusions from unpublished abstracts. Moreover, some critics have brought up concerns that many conferences have increased numbers of accepted abstracts, resulting in lesser quality research being presented, with most clinical research not being very useful.^32^ This potential decrease in conference abstract quality may explain the low publication rate among poster abstracts throughout medical specialties. Given our findings, conference and society leaders may reconsider their abstract acceptance threshold and number of accepted studies to hopefully improve the subsequent conversion to publication rate.

The reason studies remain unpublished is not well understood, but work suggests some possible reasons include studies with negative findings, projects unable to withstand rigorous peer-review, lack of time to formulate a manuscript, uncompleted projects, or manuscripts still being written.^33^ Although one study in 2003 surveyed medical researchers to determine barriers to publication, determining the current barriers for publication would allow improvements in the pathways for quality dissemination of research.^33^ For example, given the documented phenomenon of publication bias, the creation of the “research letter” or “short communication” has increased the publication opportunity for studies with preliminary, pilot, or negative findings.^34^ Each of the subspecialty societies included in our analysis has an associated journal with a fast-track conference publication option. Because fewer than 30% of abstracts are published in these society associated journals, optimizing mechanisms to improve dissemination along these pathways may be worthwhile. Additionally, among subspecialty societies, only AUGS requires a full manuscript submission for oral presentations. Whether this requirement improves dissemination remains unclear.

Furthermore, specifically determining and understanding the barriers to publication as experienced by junior researchers and medical trainees would provide significant insight. In our current study, we collected metrics on the first author and last authors’ currently held highest degree, but we do not have the capacity to determine whether an author was a trainee at the time of the conference presentation. The relationship between publication outcomes among medical or research trainees remains unknown; if a correlation is present, interventions can be targeted toward workshops or seminars to lessen this knowledge gap.

Our study has limitations. First, our query was limited to English journals indexed in PubMed, which may have missed some publications. We used PubMed because it is considered the most reputable and most often utilized database and all the subspecialty society journals were indexed there at the time of this study. Inclusion of an additional database may have highlighted some additional manuscripts that were converted to publication; thus, our findings may be underestimating the publication rate, although this underestimation would be consistent among subspecialty societies. Second, the abstract may have been published without inclusion of the abstract's first author, which would have resulted in the publication being missed in our collection method. Third, we are unable to comment on whether study findings were positive or negative, which has been shown to be associated with subsequent publication. Fourth, a publication delay may be within the realm of normal for basic science studies rather than other clinical research types. Fifth, associated funding sources often are available for published studies but not readily available for conference abstracts or unpublished studies. Finally, if authors had changes in their names from 2019 to the present time or if the research study title, keywords, or major content changed, their publication may have gone unrecognized.

Our study also has multiple strengths. First, we simultaneously analyzed the publication conversion and secondary characteristics among multiple obstetrics and gynecology subspecialty societies for a more representative assessment of women's health research dissemination and the ability to compare across different subspecialties. Second, we included both oral and poster abstracts. Third, we allowed a 5-year period for publication conversion, which is considered sufficient given prior studies in the literature. Finally, we used a multimodal systematic search on PubMed to determine publication.

Our contemporary evaluation of women's health research dissemination shows that a significant portion of research presented at obstetrics and gynecology subspecialty societies remains unpublished even after 5 years. There are also significant differences between publication metrics among each subspecialty society. Our findings highlight the need to improve the mechanism for or access by which highly regarded research translates to publication.
